# Short-term outcomes and inflammatory stress response following laparoscopy or robotic-assisted transabdominal preperitoneal inguinal hernia repair (TAPP): study protocol for a prospective, randomized trial (ROLAIS)

**DOI:** 10.1186/s13063-024-08361-w

**Published:** 2024-08-08

**Authors:** Alexandros Valsamidis Valorenzos, Kristian Als Nielsen, Karsten Kaiser, Per Helligsø, Mark Bremholm Ellebæk, Allan Dorfelt, Sofie Ronja Petersen, Andreas Kristian Pedersen, Michael Festersen Nielsen

**Affiliations:** 1 Department of General Surgery, University Hospital of Southern Denmark, Aabenraa, Denmark; 2 Department of Gynecology and Obstetrics, University Hospital of Southern Denmark, Aabenraa, Denmark; 3 Research Unit of Surgery, Odense University Hospital, Odense, Denmark; 4 Department of Clinical Research, University of Southern Denmark, Odense, Denmark; 5 Department of General Surgery, Odense University Hospital, Odense, Denmark; 6 Institute of Regional Health Research, University of Southern Denmark, Aabenraa, Denmark; 7 Department of Clinical Research, University Hospital of Southern Denmark, Aabenraa, Denmark

**Keywords:** Robotic surgical procedures, Hernia, Inguinal, Pathological conditions, Anatomical

## Abstract

**Background:**

Inguinal hernia repair is a frequently performed surgical procedure, with laparoscopic repair emerging as the preferred approach due to its lower complication rate and faster recovery compared to open repair. Mesh-based tension-free repair is the gold standard for both methods. In recent years, robotic hernia repair has been introduced as an alternative to laparoscopic repair, offering advantages such as decreased postoperative pain and improved ergonomics.

This study aims to compare the short- and long-term outcomes, including the surgical stress response, postoperative complications, quality of life, and sexual function, between robotic-assisted transabdominal preperitoneal (rTAPP) and laparoscopic TAPP inguinal hernia repairs.

**Methods:**

This randomized controlled trial will involve 150 patients from the Surgical Department of the University Hospital of Southern Denmark, randomized to undergo either rTAPP or laparoscopic TAPP. Surgical stress will be quantified by measuring C-reactive protein (CRP) and cytokine levels. Secondary outcomes include complication rates, quality of life, sexual function, and operative times. Data analysis will adhere to the intention-to-treat principle and will be conducted once all patient data are collected, with outcomes assessed at various postoperative intervals.

**Discussion:**

This study holds significance in evaluating the potential advantages of robotic-assisted surgery in the context of inguinal hernia repairs.

It is hypothesized that rTAPP will result in a lower surgical stress response and potentially lower the risk of postoperative complications compared to conventional laparoscopic TAPP. The implications of this research could influence future surgical practices and guidelines, with a focus on patient recovery and healthcare costs. The findings of this study will contribute to the ongoing discourse surrounding the utilization of robotic systems in surgery, potentially advocating for their broader implementation if the benefits are substantiated.

**Trial registration:**

ClinicalTrials.gov NCT05839587. Retrospectively registered on 28 February 2023.

## Administrative information

Note: the numbers in curly brackets in this protocol refer to SPIRIT checklist item numbers. The order of the items has been modified to group similar items (see http://www.equator-network.org/reporting-guidelines/spirit-2013-statement-defining-standard-protocol-items-for-clinical-trials/).

**Table:** 

Title {1}	Short-term outcomes and inflammatory stress response following laparoscopy or robotic-assisted transabdominal preperitoneal inguinal hernia repair (TAPP): study protocol for a prospective, randomized trial (ROLAIS)
Trial registration {2a and 2b}.	ClinicalTrials.gov ID: NCT05839587
Protocol version {3}	Version: 1.3
Funding {4}	This study is funded by the University Hospital of Southern Denmark and the Department of Regional Health Research.
Author details {5a}	^1^ Department of General Surgery, University Hospital of Southern Denmark ^2^ Department of Gynecology and Obstetrics, University Hospital of Southern Denmark ^3^ Research Unit of Surgery, Odense University Hospital, Odense, Denmark ^4^ Department of Clinical Research, University of Southern Denmark ^5^ Department of General Surgery, Odense University Hospital ^6^ Institute of regional health research, University of southern Denmark ^7^ Department of Clinical Research, University Hospital of Southern Denmark
Name and contact information for the trial sponsor {5b}	University Hospital of Southern Denmark Kresten Philipsens Vej 15, DK 6200 Phone: + 45 7997 0000 Department of Regional Health Research – University of Southern Denmark Campusvej 55, Odense M, DK 5230 Phone: + 45 6550 3828
Role of sponsor {5c}	The funding bodies had no role in the design of the study, collection, analysis, and interpretation of data, writing the manuscript or the decision to submit and will not have any authority over any of these activities.

## Introduction

### Background and rationale {6a}

Around 10,000 inguinal hernia repairs are performed annually in Denmark. Over the last decade, laparoscopic repair became the standard for primary unilateral and bilateral hernias. It shares complications with open repair but offers advantages such as reduced chronic pain, lower risk of wound infection, and faster recovery [1, 2]. Common issues include postoperative bleeding, infection, seroma, and acute/chronic pain. Recurrence rates are similar between laparoscopic and open Lichtenstein’s method, ranging between 1 and 3% [3]. Estimated chronic pain after either repair is 10%, higher with open surgery [4].

Mesh-based tension-free repair is the gold standard for both open and laparoscopic methods. Danish/international guidelines recommend laparoscopy for women, recurrence cases, where the primary hernia was repaired using an open approach, and bilateral hernias. Lichtenstein is recommended for recurrence cases, where the primary hernia was repaired using a laparoscopic method. In men, Lichtenstein or laparoscopy is chosen based on surgeon expertise, finances, and patient preference [5, 6]. Laparoscopy is generally pricier. Transabdominal preperitoneal (TAPP) and extended totally extraperitoneal (eTEP) methods are equally valid [6, 7]. TAPP is more common in Denmark.

Robotic hernia repair, introduced by Dominguez et al. in 2015, offers an alternative to laparoscopy [8]. Purported benefits include less postoperative pain, better ergonomics, and training potential [5, 9, 10]. Despite quick robotic adoption, few studies have compared short/long-term results, costs, and quality of life for laparoscopy (TAPP) versus robotic-assisted (rTAPP) methods. Conflicting data exists [11–14]; no study strongly favors robotics. Studies' limitations might have overlooked robotic benefits.

Less neurovascular trauma in the groin with rTAPP could mean less chronic pain. This could be more evident after complex hernia repairs needing advanced laparoscopic skills and thorough dissection.

Less surgical trauma correlates with lower C-Reactive Protein (CRP) and cytokine response [15, 16]. Surgery activates neuroendocrine, cytokine, acute phase, and metabolic responses tied to surgical trauma’s extent [17]. Sympathetic activation causes catecholamine release, leading to symptoms. Pituitary hormones, corticotropin, and vasopressin are also released. Proinflammatory cytokines like TNF-α, IL-8, especially IL-6, peak 1–2 h post-op. These trigger acute phase protein production (CRP) countered by anti-inflammatory cytokines like IL-10. Response’s intensity relates to surgical trauma [18–20].

This protocol gauges surgical stress by measuring CRP and cytokines. Focus is on relating surgical stress to post-op outcomes, including chronic pain.

### Objectives {7}

The primary aim of this study is to investigate whether rTAPP repair of both simple and complex inguinal hernias is associated with a lower surgical stress response, as indicated by CRP levels.

Secondarily, the study aims to assess whether rTAPP is associated with a lower cytokine response, reduced risk of postoperative complications, improved postoperative quality of life, and decreased incidence of sexual dysfunction when compared to conventional laparoscopic TAPP.

We hypothesize that the level of CRP in patients undergoing r-TAPP is lower than in patients undergoing TAPP.

The following objectives will be explored:

1. Is the systemic inflammatory response lower in rTAPP compared to TAPP measured by CRP and cytokine levels in serum?
2. Is the risk of short-term and long-term complications different when comparing rTAPP to TAPP?
3. Is postoperative quality of life different when comparing rTAPP to TAPP?
4. Is postoperative sexual function different when comparing rTAPP to TAPP?
5. Is the effective operative time different when comparing rTAPP to TAPP?

### Trial design {8}

The study will be a single-center, open-label, randomized controlled trial (parallel-group) (1:1 allocation) comparing TAPP and r-TAPP using data from patients operated at the Surgical Department of the University Hospital of Southern Denmark.

The study will follow a superiority study design aiming to investigate whether rTAPP of complex inguinal hernias is associated with a lower surgical stress response and a lower risk of postoperative complications compared to laparoscopic TAPP.

## Methods: participants, interventions, and outcomes

### Study setting {9}

Participants will be recruited at the outpatient clinic of the Surgical Department of the University Hospital of Southern Denmark, Aabenraa.

### Eligibility criteria {10}

#### Inclusion criteria

1. Age ≥ 18
2. ASA 1–3
3. Clinical or radiologic diagnosis of inguinal hernia
  a Unilateral inguinal hernia
  b Bilateral inguinal hernias
  c Recurrent inguinal hernia
  d Inguinoscrotal hernia
4. Patients will complete a preoperative anesthesiologic assessment and must be eligible for a laparoscopic procedure
5. Informed consent

#### Exclusion criteria

1. Incarcerated inguinal hernia requiring emergency surgery
2. Pregnancy
3. Patients with chronic pain due to arthritis, migraine, or other illnesses requiring regular intake of pain relievers (paracetamol, NSAID, etc.).
4. Co-existing cancer
5. History of psychiatric or additive disorder that prevents the patient from participating in the trial
6. Insufficient Danish language proficiency
7. Co-existing inflammatory disease
8. Co-existing immunological disease that requires medication of any kind

#### Individuals who will perform the intervention

Four experienced surgeons who perform both procedures routinely will perform all interventions.

### Who will take informed consent? {26a}

**Fig. 1:**
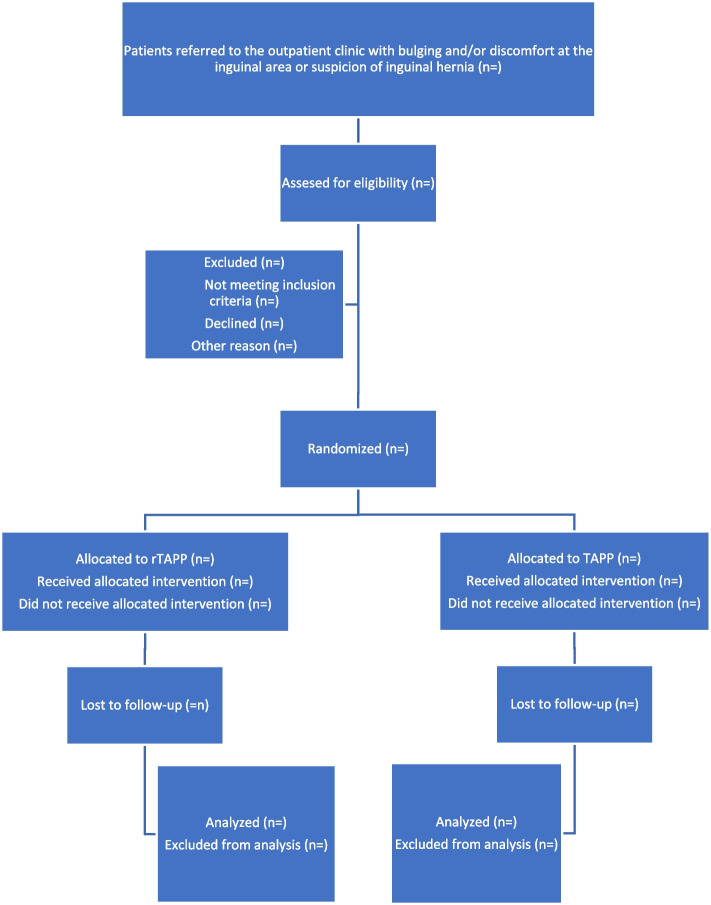
Flowchart

Participants will be recruited at the outpatient clinic. If there is an indication for surgery, they will be informed about the opportunity of participating in the study and provided with informational material about the study. The primary investigator will then confirm eligibility and the patients will be asked to provide written informed consent by the primary investigator. Consent can be withdrawn at any point during the trial. The flowchart is shown in Fig. 1.

### Additional consent provisions for collection and use of participant data and biological specimens {26b}

No additional studies are planned and consent will not be obtained for that potentiality.

## Interventions

### Explanation for the choice of comparators {6b}

The comparator, conventional laparoscopic TAPP, has been chosen due to its established role as the current standard of care for inguinal hernia repair in many surgical centers worldwide, including Denmark. Laparoscopic TAPP is recognized for its advantages over open surgery, such as reduced postoperative pain, lower incidence of wound infection, and faster recovery, making it a gold standard against which new surgical techniques should be measured. The widespread adoption and extensive clinical experience with laparoscopic TAPP provide a solid foundation for comparison, ensuring that the trial's findings are relevant and can inform clinical practice effectively.

Robotic-assisted surgery has emerged as a promising alternative to traditional laparoscopic approaches, offering potential benefits such as improved ergonomics for the surgeon, greater precision during the procedure, and possibly reduced postoperative pain for patients. However, the evidence base supporting its superiority or equivalence to laparoscopic TAPP, particularly in terms of surgical stress response and postoperative recovery, remains inconclusive. By comparing rTAPP directly with the well-established TAPP method, this trial aims to address these gaps in the literature, providing high-quality evidence on the comparative efficacy and safety of these two approaches.

The choice of comparators reflects a commitment to advancing surgical practice by rigorously evaluating the potential benefits of newer robotic technologies against the current standard of laparoscopic surgery. This comparison is crucial for understanding whether the increased costs associated with robotic surgery are justified by improved patient outcomes or whether laparoscopic TAPP continues to represent the best option for patients requiring inguinal hernia repair.

### Intervention description {11a}

#### Robotic-assisted transabdominal preperitoneal (rTAPP) intervention

The rTAPP procedure will be performed using the Da Vinci Xi Robotic Surgical System. The patient will be positioned supine on the operating table. Under general anesthesia, an 8-mm camera port will be inserted at the umbilicus, and two 8-mm robotic working ports will be placed laterally to the rectus muscles, all under direct vision. The robot will then be docked, with its arms aligned with the working ports. The peritoneal cavity will be insufflated with CO_2_ to a pressure of 12 mmHg to create a working space.

The surgeon, seated at the robotic console, will manipulate the robotic arms to incise the peritoneum and expose the preperitoneal space. Hernia sac dissection, reduction, and placement of a mesh to cover the hernia defect will be performed under robotic guidance. A ProGrip TM Self-fixating mesh will be cut to the appropriate size and placed. Finally, the peritoneum will be closed over the mesh with barbed absorbable sutures with the abdominal pressure set to 8 mmHg. The robot will be undocked, and the ports removed. The skin at the incision sites will be closed using absorbable sutures.

#### Conventional laparoscopic TAPP intervention

The TAPP procedure will be performed similarly to the robotic technique. The patient will be positioned in a similar manner to the rTAPP group. Under general anesthesia, a 12-mm camera port will be placed at the umbilicus, and two 5-mm working ports will be inserted laterally into the rectus muscles. CO2 insufflation will be used to create a pneumoperitoneum with a pressure of 12 mmHg.

The laparoscopic surgeon will perform the hernia repair using a laparoscope and standard laparoscopic instruments. This involves incising the peritoneum, dissecting the hernia sac, reducing the hernia, and placing a ProGrip TM Self-fixating mesh, cut to the appropriate size, over the defect. The peritoneum will then be closed over the mesh using barbed absorbable sutures. The ports will be removed, and the incisions closed in layers, with the skin closed using absorbable sutures.

#### Administration and timing

Both interventions will be administered under general anesthesia, with patients fasting for at least 6 h prior to the procedure. No antibiotic prophylaxis and no corticosteroids will be administrated. Pain management will be standardized across both groups, with patients receiving a combination of systemic analgesics and local anesthetic infiltration at the port sites. This will not include the administration of Non-Steroid Anti-Inflammatory Drugs (NSAID).

Patients will be admitted on the day of surgery and are expected to be discharged the same day, subject to their recovery and clinical criteria. Follow-up visits will be scheduled at 1 day and 3 days postoperatively to take blood tests.

### Criteria for discontinuing or modifying allocated interventions {11b}

Based on clinical judgment, the surgeon may decide to discontinue or modify an intervention if deemed in the best interest of the participant.

Should technical difficulties arise during the procedure or unexpected intraoperative findings necessitate a change in surgical approach (e.g., conversion from rTAPP/TAPP to an open procedure), the allocated intervention may be modified accordingly. This ensures the participant's safety and the best possible surgical outcome.

All modifications or discontinuations will be reported.

### Strategies to improve adherence to interventions {11c}

#### Preoperative education

Participants will receive comprehensive preoperative education, including detailed explanations of the procedures, the importance of following the postoperative care plan, and the significance of their adherence to the study protocol for the trial's success. This education will be delivered through individual consultations and written materials tailored to enhance understanding and engagement.

#### Accessibility to the research team

Participants will have direct access to the research team via phone or email to ask questions, report issues, or seek advice related to their postoperative care and adherence to the study protocol. This open line of communication ensures participants feel supported and can promptly address any barriers to adherence.

#### Reminders

It is expected that some participants might not respond to the post-operative questionnaires due to forgetfulness. To avoid this, patients will receive automated reminders in written form twice; otherwise, they will be given a phone call to ensure sufficient data coverage.

### Relevant concomitant care permitted or prohibited during the trial {11d}

#### Prohibited concomitant care

##### Perioperative corticosteroids

The use of perioperative corticosteroids is strictly prohibited. Corticosteroids have well-documented effects on reducing inflammation and could significantly alter the levels of CRP and interleukins in the bloodstream, thereby confounding the trial’s outcomes.

##### NSAIDs

Participants are not allowed to receive NSAIDs perioperatively. NSAIDs are known to exert anti-inflammatory effects, which could impact the measurement of inflammatory markers, including CRP and interleukins. This prohibition ensures the accuracy of the trial’s inflammatory response assessment.

#### Permitted concomitant care

##### Standard postoperative analgesia and antiemetics

Participants will be allowed to receive standard postoperative analgesia and antiemetics that do not include NSAIDs or corticosteroids. This may include ondansetron, paracetamol, and opioid analgesics, which do not significantly affect CRP and interleukin levels, to manage postoperative pain and nausea effectively.

##### Antibiotics

Prophylactic and therapeutic use of antibiotics, as per local guidelines to prevent and treat surgical site infections, is permitted if the clinician deems it necessary.

### Provisions for post-trial care {30}

Participants will have access to follow-up care after the completion of the trial to monitor and address any long-term effects or complications arising from the surgical interventions.

All study participants will be covered by the Danish Patient Compensation Association (“Patienterstatningen”).

### Outcomes {12}

#### Exposure and adjustment variables

The exposure variable will be a binary variable describing whether the patient received conventional laparoscopic surgery or robotic-assisted surgery.

In cases where the link function of the statistical model is other than the identity, strong outcome predictors are adjusted to achieve the collapsibility of results [21].

#### Outcome variables (outcome definition)

##### CRP (primary outcome)

Level of plasma CRP measured in mg/L (continuous), change from baseline to 1 and 3 days after operation.

##### Secondary outcomes

Secondary outcomes include the following:

a Inflammatory variables (ng/L), continuous, change from baseline to 30 min and 120 after extubation. The difference between the baseline and the post-surgical value will be indicated
  i. IL-1β
  ii. IL-6
  iii. IL-8
  iv. IL-10
  v. TNF-α
b Intra and postoperative outcomes are going to be measured at the end of surgery
  i. Level of required blood transfusion (mL), mean (SD), continuous
  ii. Estimated intra-operative blood loss (mL), mean (SD), continuous
  iii. Hernia defect size measured at 8 mmHg (cm^2^), mean (SD), continuous
  iv. Postoperative length of stay (overnight stay yes or no), categorical
  v. Total surgical time (minutes), median (IQR) discrete
  vi. Total anesthesia time (minutes), median (IQR) discrete
  vii. Postoperative analgesis, n (%), binary
  viii. Any postoperative complications, binary
    1. surgical complications, n (%)
      Haematoma, seroma, wound infection, hernia recurrence, pain.
    2. Medical complications, *n* (%), binary
      Cerebral stroke, STEMI/NON-STEMI, aspiration, pneumonia, heart insufficiency, pulmonary embolism, respiratory failure, renal insufficiency, sepsis, deep vein thrombosis, arterial thromboembolism
  ix. Clavien-Dindo Classification
    Five levels according to the grading scale. If a patient has multiple complications, the highest grade is indicated.
c Questionnaires, ordinal, measured at baseline,
  i. European registry for abdominal wall hernias quality of life score (EuraHS-QoL) (baseline, 1 month post, 3 months, and 6 months) measured on a scale from 0 (highest life quality) to 180 (lowest life quality)
  ii. Questionnaire for inguinal hernia pain related sexual dysfunction (SexIHQ) (baseline, 6 months). The first question identifies “sexually active” patients. The second identifies patients having *No pain during sexual activity* (NPS) or *Pain during sexual activity* (PS). Only patients with *Pain during sexual activity* proceed to the following questions; assessing pain-induced sexual activity impairment (yes/no), pain frequency (rarely/often/always), pain intensity (VAS, 0–10) erectile dysfunction (VAS, 0–10), ejaculatory dysfunction (VAS, 0–10) and self-perceived feeling of depression (yes/no).
d Surgical time in minutes, median (IQR), discrete
  i. Part 1
  ii. Part 2
  iii. Part 3
  iv. Part 4

The procedure will be divided into 4 parts. Part 1 will be different for the 2 procedures. In rTAPP, it will consist of docking of the robot and port placement while it only will consist of port placement in TAPP. Parts 2 and 3 will be the same for both procedures and will consist of hernia reduction and preparation of the preperitoneal space where the mesh is placed (part 2), mesh placement, and suturing of the peritoneum (part 3). Part 4 will also be different for the 2 procedures. In rTAPP, it will consist of de-docking and skin closure while it only will consist of skin closure in TAPP. Total surgical time and each part will be measured individually in minutes and the 2 procedures will be compared.

Potential harms are listed as secondary outcomes and are analyzed in the respective part.

### Participant timeline {13}

Participants will be recruited from the outpatient clinic. In cases where surgery is indicated, they will be informed about the possibility of participating in the study and provided with informational materials. Eligibility will be confirmed by the primary investigator, who will then request written informed consent from the patients.

#### Preoperative phase

Enrolled participants will be required to complete preoperative questionnaires.

Preoperative blood samples will be collected.

Baseline demographic data will also be gathered.

#### Perioperative phase

Data will be collected on the day of surgery.

Blood samples will be taken 30 min and 120 min after extubation.

#### Postoperative phase

Additional blood tests will be conducted on days 1 and 3 following the surgery.

Participants will complete the EuraHS-QoL questionnaire at 1, 3, and 6 months post-surgery.

The SexIHQ questionnaire will be administered 6 months after surgery.

Postoperative outcomes will be continuously collected from the end of the surgery until the trial’s conclusion.

**Figure:**
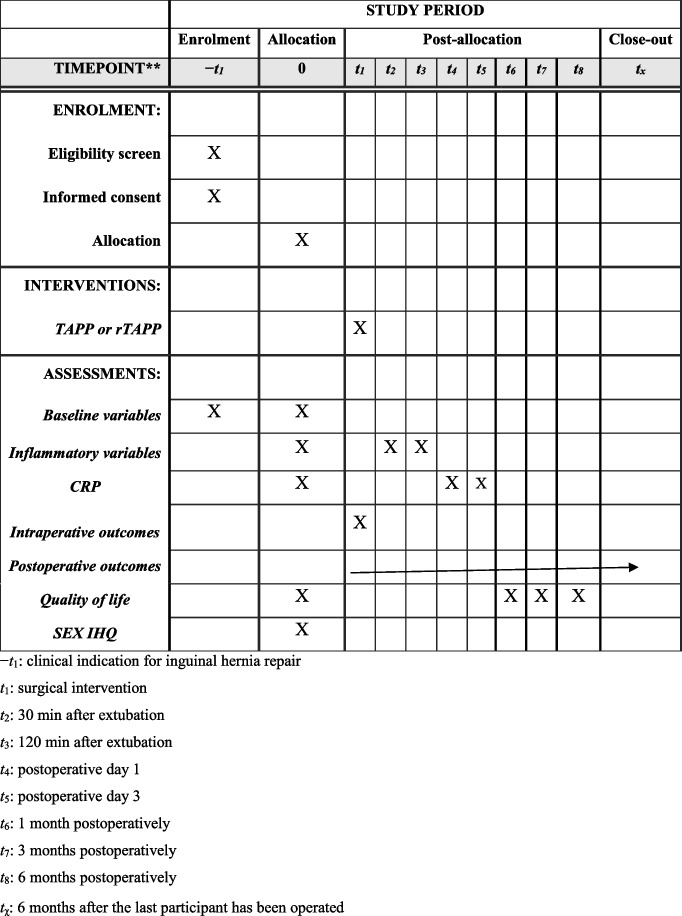
Schematic presentation of study’s timeline

### Sample size {14}

A Monte Carlo simulation was employed to calculate the power of the mixed-effect model utilized for the log-normally distributed outcome, CRP. No power calculations were performed for other outcomes. The sample size was determined based on the premise that the CRP levels in patients undergoing r-TAPP were anticipated to be 15% lower compared to those undergoing traditional TAPP surgery. This assumption was grounded on a study from 2019, which reported an average CRP value of 16.5 mg/L 24 h post-TAPP surgery for inguinal hernia repair [22]. Additionally, an observational study with data from 298 patients who underwent surgery for colon cancer with either a robotic-assisted or a laparoscopic approach between 2017 and 2019 at the Surgical Department of the Hospital of Southern Jutland informed this assumption [15]. The latter study observed that CRP levels measured on the third postoperative day were 28% lower in patients who received the robotic-assisted procedure compared to those who underwent the laparoscopic method. Given that inguinal hernia repair is less invasive than colonic resection, a smaller difference in CRP levels was anticipated. Consequently, a 15% difference was adopted for the basis of our calculations. With a cohort of 150 patients—112 presenting uncomplicated cases and 38 complicated—we achieved a statistical power of 90% for the complicated group and 80% for the uncomplicated group.

### Recruitment {15}

Recruitment will be conducted continuously among eligible patients until the target sample size is achieved. Participants will be recruited among patients at the outpatient clinic. If there is indication for surgery, they will be informed about the opportunity of participating in the study and provided with informational material about the study. The primary investigator will then confirm eligibility and the patients will be asked to provide written informed consent which can be withdrawn at any point during the trial.

## Assignment of interventions: allocation

### Sequence generation {16a}

Following recruitment, enrolled patients are randomly assigned to one of the two study arms, i.e., robot-assisted or laparoscopic inguinal hernia repair.

Patients will be randomized on a 1:1 basis using a computer-generated randomization sequence without stratification using blocks of 6.

### Concealment mechanism {16b}

The randomization tool in Research Electronic Data Capture (REDCap) will be used.

### Implementation {16c}

The randomization sequence will be programmed by an independent data manager. The project manager and assistants administering the allocation will not have access to the randomization code.

## Assignment of interventions: blinding

### Who will be blinded {17a}

The study is not blinded.

### Procedure for unblinding if needed {17b}

The design is open label with only outcome assessors being blinded so unblinding will not occur.

## Data collection and management

### Plans for assessment and collection of outcomes {18a}

#### Clinical and laboratory assessments

##### CRP

The primary outcome measure, CRP, will be quantified using blood samples collected at three time points: baseline, 1 day, and 3 days post-operatively. These laboratory tests will be conducted at the central lab of the University Hospital of Southern Denmark, utilizing standardized procedures to ensure consistency and reliability across all measurements.

##### Cytokines

The secondary outcome measures, including cytokine profiles (IL-1β, IL-6, IL-8, IL-10, and TNF-α), will be quantified using blood samples. These samples will be collected at baseline, 30 min, and 120 min post-operation and stored in a biobank. After the collection of the last blood sample from the last participant, these samples will be transported to the neurobiological department of the University of Southern Denmark for analysis. To verify results and ensure accuracy, duplicate measurements will be performed.

#### Surgical and postoperative outcomes

Data on intraoperative blood loss, hernia defect size, mesh size, surgical and anesthesia time, and postoperative complications will be systematically documented using structured forms. The Clavien-Dindo Classification will be applied to categorize and assess the severity of surgical complications.

#### Questionnaires

Quality of Life and Sexual Dysfunction: To assess patients’ quality of life and sexual function, the EuraHS-QoL and the SexIHQ questionnaire will be utilized. REDCap will automatically send these questionnaires at predetermined time points. The EuraHS-QoL, previously validated, will be distributed at baseline, 1 month, 3 months, and 6 months postoperatively. The SexIHQ will be sent at baseline and 6 months postoperatively.

#### Data collection forms

All data will be collected using standardized forms specifically developed for this study in REDCap, which are available upon request from the study team. Furthermore, REDCap will be configured to include range checks for data values, aiming to minimize errors during data entry and enhance the quality of the data collected.

### Plans to promote participant retention and complete follow-up {18b}

#### Regular communication

Maintaining open and frequent communication with participants through various means, such as emails, SMS reminders, and phone calls, will be a priority. Automated SMS reminders will be sent to remind participants of their upcoming scheduled blood tests. Additionally, to minimize forgetfulness regarding questionnaire completion, participants will receive email reminders twice; if there is no response, a follow-up phone call will be made to ensure complete data collection.

#### Flexible scheduling

Recognizing that participants have different personal and professional commitments, the study will offer flexible scheduling for of the time at which the participants can get their blood tests taken on the predetermined postoperative days. By accommodating participants' schedules, the likelihood of complete follow-up is increased.

#### Participant support

The main investigator will be directly accessible to participants for any queries or concerns throughout the study. Contact information, including the primary investigator's phone number, will be provided to all participants. This support system aims to build trust and rapport with participants, making them feel valued and supported.

#### Travel reimbursement and incentives

To alleviate the potential burden of travel costs, participants will be reimbursed for travel expenses related to study visits.

#### Handling discontinuations and deviations

For participants who discontinue or deviate from the intervention protocols, efforts will be made to collect outcome data as per the original study timeline, ensuring that data analysis can be as comprehensive as possible.

#### Reasons for discontinuation/deviation

Information on why a participant discontinued or deviated from the protocol will be collected, when possible.

### Data management {19}

The trial will utilize the REDCap platform for all data management activities.

Access to the REDCap system will be restricted through individual login credentials, assigned based on roles and responsibilities within the trial team. This ensures that only authorized personnel can access or modify the data.

Key data fields, especially those critical to the study outcomes, will be subjected to double data entry to ensure accuracy. Discrepancies will be flagged for review and correction by the data management team. REDCap will furthermore be configured to include range checks for data values to minimize errors during data entry. This includes setting acceptable ranges for numerical data and validation rules for other types of data.

### Confidentiality {27}

This study will comply to all data protection regulations and the Data Protection Act in the processing of personal data in the study. This is the responsibility of the main investigator. All study-related information will be stored in REDCap which is encrypted to protect confidentiality. REDCap's server infrastructure is designed to ensure secure data storage and backup. Each trial participant will be assigned a unique identifier (ID) to ensure confidentiality. All data collected will be associated with this ID rather than personal identifying information. All blood samples will also be identified by a coded ID and stored securely at an onsite biobank.

### Plans for collection, laboratory evaluation, and storage of biological specimens for genetic or molecular analysis in this trial/future use {33}

Blood samples will be obtained preoperatively, on the day of admission, 30 and 120 min after arrival in the recovery room following the procedure, and on postoperative days 1 and 3. Serum for analysis will collected in NUC vails and stored at − 80 °C. The cytokine panel to be measured will consist of CRP, IL-1β, IL-6, IL-8, IL-10, and TNF-α. CRP will be measured preoperatively and postoperatively on days 1 and 3. The secondary cytokine profiles will be collected preoperatively and postoperatively 30 min and 120 min after extubation.

Ten milliliters venous blood will be taken in separator gel collection tubes, after which the samples will be centrifuged. The total amount of blood taken per patient during the course of the study will be 50 mL. The serum will be collected and transferred to cryotubes and immediately frozen to − 80 °C. The samples will be stored in a biobank with unique identification numbers. When all study samples have been collected (assumed to be completed after 24 months), the vials will be transferred to freezer boxes containing dry ice and transported to the neurobiological department, University of Southern Denmark for analysis. All samples will be destroyed after the analyses have been performed and will not be stored for future research.

## Statistical methods

### Statistical methods for primary and secondary outcomes {20a}

#### Descriptive statistics

Descriptive statistics will be utilized in order to check for exchangeability between the two groups of operation according to the baseline variables. Categorical variables will be analyzed with a chi-square test or Fischer’s exact test depending on Cochran’s rule [23]. Continuous and discrete variables will be analyzed with an independent *t*-test or Wilcoxon rank-sum test depending on the distribution of the variable. The variables will be presented as highlighted in the baseline table.

Besides the descriptive tables, we will also present margins and spaghetti plots.

#### Main analysis

A linear mixed effect model will be applied to analyze the difference in CRP level over time between the two study groups. The model will control for the interpersonal variation of the participants, random slope, and the potential effect modification by time and difference at baseline. As the treatment effect varies over time a likelihood ratio test will be conducted to test whether there is statistically significant treatment across all timepoints [24].

The model control will consist of a graphical assessment of the normality of the fitted values of the random effects and the residuals.

If the fit of the mixed is not satisfactory, either a log transformation of the outcome will be conducted or we will use a bootstrapped likelihood ratio test.

We will also present a margins plot based on the model and compare it with the margins plots conducted in the descriptive analysis.

#### Supplementary analyses

- Change in inflammatory variables (interleukins and TNF-α) are tested for an overall difference with linear mixed effect models for the abovementioned reasons. Model control and parametrization of the model will be similar to the analysis of the CRP.
- Intra- and postoperative outcomes
  xxii. Level of required blood transfusion, estimated intra-operative blood loss, hernia defect size, total surgical time, and total anesthesia time will be tested via a two-sample *t*-test or a rank-sum test in case of normality violations.
  xxiii. Whether the patient stayed overnight will be analyzed with logistic regression or by way of Fischer’s exact test depending on the number of cases or non-cases.
  xxiv. The difference in the proportion of patients that require postoperative analgesics is evaluated via a logistic regression or Fischer’s exact test depending on the number of cases. The same applies to postoperative and medical complications.
  xxv. Clavien-Dindo will be evaluated by way of either χ2 test or Fischer’s exact test.
- Quality of life is expressed as overall score values with a natural minimum and maximum value. To accommodate the repeated measurements, linear mixed effect beta-binomial regression will be applied again. As the difference between quality of life can vary between the two groups over time, we will again conduct a likelihood ratio test to assess if there is a statistically significant treatment effect across all timepoints. In order to estimate the difference between the two operation groups for each time point, we will utilize G-computation.
- Sexual dysfunction will be purely descriptive. Non-categorical variables will be either presented with mean and standard deviation or median and interquartile range, depending on whether the normal distribution is fulfilled or not. Categorical variables will be presented with numbers and percentages.
- The difference in surgical time per part (1–4) is assessed using separate rank-sum tests and the median times.

#### Statistical software

Statistical analyses will be performed using Stata and R. We will use the JM package when we conduct the joint modeling.

### Interim analyses {21b}

No interim analyses will be conducted, and there will be no formal stopping rules for this study. The two procedures under comparison—TAPP and r-TAPP—are widely established with well-documented efficacy. Given their established clinical utility, the primary focus of our study is not to establish efficacy but to compare nuanced differences in outcomes such as surgical stress response.

Both procedures are widely considered safe, with known and manageable risks, and neither procedure is currently considered superior to the other. Furthermore, in the department's current setup, patients would receive one of the two procedures at random, based on which procedure had the shortest waiting time at the time when the indication for surgery was established. Consequently, the likelihood of encountering unexpected safety issues requiring the implementation of formal stopping rules, from an ethical standpoint, is low.

Nevertheless, continuous monitoring of adverse events will be rigorously conducted throughout the trial. This ensures that any significant safety concerns are promptly addressed and reported to the ethical committee, adhering to all regulatory requirements.

Lastly, conducting interim analyses can often lead to increased risks of type I errors. By avoiding interim analyses, we maintain the full statistical power intended for the conclusive end-of-study analysis. This ensures that our findings are both robust and reliable. This approach avoids the complexities of statistical adjustments associated with multiple analysis points, focusing on delivering clear, comprehensive conclusions at the study’s completion.

### Methods for additional analyses (e.g., subgroup analyses) {20b}

To assess whether “complicated cases” are an effect modifier, we will stratify the main and secondary analyses for this factor to see whether there is a significant interaction term based on a likelihood ratio test.

### Methods in analysis to handle protocol non-adherence and any statistical methods to handle missing data {20c}

It is expected that there will be a low level of withdrawal from the study. The study will include people able to benefit of this operation, therefore postoperative and perioperative outcomes will be unlikely to be missing, and a complete case analysis will be adhered to.

Missing data includes patient withdrawals, blood samples that are not possible to collect, and failure of the patients to return the questionnaires. Missing data will be assumed to be missing at random for the primary analysis and no multiple imputation will be utilized due to the maximum likelihood estimation that is used in generalized linear mixed models.

In relation to the outcomes relating to quality of life and pain, we will assume the data to be missing not at random, and therefore use joint modeling, where we will use cox-regression to model the missing data mechanism.

### Plans to give access to the full protocol, participant-level data and statistical code {31c}

Trial data can be made available upon request.

## Oversight and monitoring

### Composition of the coordinating center and trial steering committee {5d}

The Coordinating Centre, which also functions as the Project Management Group, is responsible for the comprehensive administration and daily management of the trial. This team oversees crucial aspects such as participant recruitment, data collection, and regulatory compliance, ensuring that trial operations adhere strictly to the protocol and meet ethical standards. The group meets bi-weekly to review and manage detailed aspects of the trial's day-to-day operations, including monitoring recruitment rates, ensuring the consistency of data collection, and efficiently allocating resources. This integrated approach helps maintain the organizational structure and supports the broader goals set by the Trial Steering Committee, ensuring the trial’s success and integrity.

The Trial Steering Committee, which includes the primary investigator, two additional investigators, and key stakeholders from the host institution, meets monthly. This committee’s responsibilities extend beyond general oversight to include a detailed review of trial progress, efficacy data, participant safety, and adherence to protocols. They also address and resolve any operational issues that arise, ensuring the trial’s integrity and timely progress.

### Composition of the data monitoring committee, its role and reporting structure {21a}

Given the low-risk nature of the intervention, a formal Data Monitoring Committee was not established. Instead, the primary investigator functions as the data manager, overseeing the integrity and security of the trial data. Continuous monitoring of data quality and participant safety is maintained through regular audits conducted by the Ethics Committee and ad hoc reviews by the Trial Steering Committee.

Regular access to trial data is granted only to authorized personnel within the coordinating center, ensuring compliance with regulatory requirements and maintaining data confidentiality. The Ethics Board may request access to data as part of its auditing and oversight role, ensuring continuous compliance with ethical standards.

### Adverse event reporting and harms {22}

Patients included in the study would undergo the same surgical procedure, regardless of this study. Both procedures have the same expected risks and complications. Both surgical procedures are widely used internationally, and it is expected that the majority of the potential complications may be termed as expected. The known complications of elective hernia repairs, that are not directly caused by this study can be divided into surgical (peri- and postoperative), medical (peri- and postoperative), anesthesiologic, and technical caused by the equipment. In the event that several unexpected complications occur during the trial, the study will be interrupted. Complications can be described as being serious and unexpected in the event of (1) death or (2) life-threatening condition.

Of the expected perioperative surgical complications, the following can be mentioned:

- Bleeding
- Intraoperative lesions (organs and vascular structures)
- Fecal contamination
- Failure of surgical equipment

The following can also be expected in postoperative surgical complications

- Bleeding
- Fascial dehiscence
- Surgical site infection
- Intraabdominal abscess formation

The following can also be expected in postoperative medical complications

- Cerebrovascular stroke or hemorrhage
- STEMI/NON-STEMI
- Cardiac arrhythmia
- Aspiration
- Pneumonia
- Heart insufficiency
- Deep vein thrombosis
- Pulmonary embolism
- Respiratory insufficiency
- Renal insufficiency
- Sepsis
- Arterial embolism
- Urinary tract infection (lower and upper)
- Urinary retention

Adverse events are summarized and analyzed in the respective separate analyses. All adverse events will be reported. 

### Frequency and plans for auditing trial conduct {23}

Audits of the trial conduct are scheduled to occur semi-annually and are conducted by the Ethics Committee. These audits are designed to verify adherence to the trial protocol and ethical guidelines, assessing elements such as consent process, data management practices, and overall trial management. Findings from these audits are reported directly to the Trial Steering Committee, which is tasked with addressing any identified issues promptly.

### Plans for communicating important protocol amendments to relevant parties (e.g., trial participants, ethical committees) {25}

Upon receiving approval from the local ethics board, any modifications to the study protocol will be officially documented as amendments on ClinicalTrials.gov to ensure transparency and regulatory compliance. The process for communicating these amendments to relevant parties, including trial participants and ethical committees, is outlined as follows:

Notification of Participants: All participants who have signed consent forms prior to any change in the protocol will be notified via email about the details of the amendment. This notification will include a clear explanation of the change, its rationale, and its potential impact on their participation in the trial. Participants will be offered the opportunity to review and sign a new written consent form if the amendment affects their rights, safety, or wellbeing.

Criteria for Participant Notification: Participants will be informed of amendments that could impact their involvement in the study or alter the risk/benefit ratio of their participation. This includes, but is not limited to, changes in study procedures, treatment schedules, or any aspect that could influence their decision to continue in the trial.

Exemptions from Participant Notification: If an amendment does not materially affect the participant's involvement or the risk/benefit ratio—such as changes in statistical methods or alterations in study personnel—participants may not be directly notified. These changes are deemed administrative and do not directly impact participants’ experience or safety within the trial.

Communication with Ethical Committees: All amendments will be promptly communicated to the ethical committees that approved the study. This ensures ongoing compliance with ethical standards and regulatory requirements. The communication will detail the amendment, its justification, and any anticipated effects on the trial conduct or participants.

### Dissemination plans {31a}

#### Publication in scientific journals

We aim to publish the outcomes of the study in reputable international peer-reviewed journals. The results will be reported in an anonymized format to protect participant confidentiality. This ensures the findings are accessible to healthcare professionals, researchers, and policymakers worldwide, facilitating the advancement of medical knowledge and practice.

#### Presentations at congresses

Findings from the study may also be presented at both national and international medical congresses. These presentations provide an opportunity for live discussion and feedback from the global medical community, promoting collaboration and potentially inspiring future research.

#### Communication to participants

In recognition of the valuable contribution of the participants to the study, they will be informed of the results via email. This communication will summarize the key findings in a manner that is understandable to laypersons, ensuring participants are aware of the outcomes of the research they contributed to.

## Discussion

The significance of this study lies in its thorough evaluation of the potential benefits associated with robotic-assisted surgery for inguinal hernia repairs, a common yet complex surgical procedure. By comparing robotic-assisted transabdominal preperitoneal (rTAPP) to conventional laparoscopic TAPP, we aim to delineate the impact of advanced robotic technology on surgical outcomes, particularly focusing on the surgical stress response and the incidence of postoperative complications.

We hypothesize that rTAPP will demonstrate a reduced surgical stress response, thereby potentially decreasing the risk of postoperative complications when juxtaposed with traditional laparoscopic approaches. Such findings could have profound implications for surgical methodology, patient care protocols, and healthcare expenditure, marking a significant step forward in the evolution of surgical practices.

The broader objective of this research is to contribute meaningfully to the current discussions on the integration of robotic systems in surgical operations. Should the anticipated benefits of robotic-assisted surgery be empirically validated, our study could serve as a pivotal advocate for the expanded use of such technologies. This, in turn, could lead to a paradigm shift in surgical standards and guidelines, emphasizing enhanced patient recovery, efficiency, and cost-effectiveness.

In summary, this study not only seeks to expand the scientific understanding of robotic-assisted surgery's merits but also aims to inform future surgical guidelines and practices, potentially heralding a new era in the surgical treatment of inguinal hernias.

## Trial status

This is protocol version: 1.3 (dated 15 January 2024).

Recruitment commenced on 11 November 2022. Recruitment is expected to end on 1 January  2025.

## Data Availability

The datasets generated and analyzed during the current study are available from the corresponding author upon reasonable request.

## Abbreviations

TAPP: Transabdominal preperitoneal hernia repair
rTAPP: Robotic-assisted transabdominal preperitoneal hernia repair
CRP: C-reactive protein
eTEP: Extended totally extraperitoneal repair
NSAID: Non-steroid anti-inflammatory drugs
EuraHS-QoL: European registry for abdominal wall hernias quality of life score
SexIHQ: Questionnaire for inguinal hernia pain related sexual dysfunction
REDCAP: Research Electronic Data Capture

## References

[1] McCormack K, Scott NW, Go PM, Ross S, Grant AM. Laparascopic techniques versus open techniques for inguinal hernia repair. Cocrane Database Syst Rev. 2003;1:CD001785. 10.1002/14651858.CD001785 PMC8407507 12535413

[2] Grant AM. Laparoscopic versus open hernia repair: meta-analysis of randomised trials based on individual patient data Hernia. 2002;6(1):2–10. 12090575 10.1007/s10029-002-0050-8

[3] Gopal SV, Warrier A. Recurrence after groin hernia repair-revisited. Int J Surg. 2013;11:374–7. 23557981 10.1016/j.ijsu.2013.03.012

[4] Kumar S, Wilson RG, Nixon SJ, Macintyre MC. Chronic pain after laparoscopic and open mesh repair after groin hernia. Br J Surg. 2002;89:1476–9. 12390395 10.1046/j.1365-2168.2002.02260.x

[5] Podolsky D, Novitsky Y. Robotic inguinal hernia repair. Surg Clin N Am. 2020;100:409–15. 32169186 10.1016/j.suc.2019.12.010

[6] Rosenberg J, Bisgaard T, Kehlet H. Danish hernia database recommendations for the management of inguinal and femoral hernia in adults Dan Med Bull. 2011;58:C4243. 21299930

[7] Siddaiah-Subramanya M, Ashrafi D, Memon B, Memon MA. Causes for recurrence in laparoscopic inguinal hernia repair. Hernia. 2018;22:975–86. 30145622 10.1007/s10029-018-1817-x

[8] Dominguez JEE, Gonzalez A, Donkor C. Robotic inguinal hernia repair. J Surg Oncol. 2015;112:310–4. 26153353 10.1002/jso.23905

[9] Edelman DS. Robotic inguinal henia repair. Surg Technol Int. 2020;28:99–104. 32432334

[10] Edelman DS. Robotic inguinal hernia repair. Am Surg. 2017;83(12):1418–21. 29336765 10.1177/000313481708301229

[11] Charles EJ, Mehaffey JH, Tache-Leon CA. Inguinal hernia repair: is there a benefit to using the robot? Surg Endosc. 2018;32:2131–6. 29067575 10.1007/s00464-017-5911-4 PMC10740385

[12] Huerta S, Timmerman C, Argo M, et al. Open, laparoscopic and robotic inguinal hernia repair: Outcomes and predictors of complications. J Surg Res. 2019;241:119–27. 31022677 10.1016/j.jss.2019.03.046

[13] Waite KE, Herman MA, Doyle PJ. Comparison of robotic versus laparoscopic transabdominal preperitoneal (TAPP) inguinal hernia repair. J Robotic Surg. 2016;10:239–44. 10.1007/s11701-016-0580-1 27112781

[14] Prabhu AS, Carbonelli A, Hope W. Robotic inguinal vs transabdominal laparoscopic inguinal hernia repair. The RIVAL randomized clinical trial JAMA. 2020;155(5):1–8. 10.1001/jamasurg.2020.0034 PMC7081145 32186683

[15] Cuk P, Simonsen RM, Komljen M, et al. Improved perioperative outcomes and reduced inflammatory stress response in malignant robot-assisted colorectal resections: a retrospective cohort study of 298 patients. World J Surg Oncol. 2021;19:155. 10.1186/s12957-021-02263-w. 34022914 10.1186/s12957-021-02263-w PMC8141231

[16] Kingo PS, Palmfeldt J, Nørregaard R. Perioperative systemic inflammatory response following robotic-asssisted laparoscopic cystectomy vs open mini-laparotomy cystectomy: a prospective study. Urol Int. 2017;99:436–45. 28668947 10.1159/000478274

[17] Moore CM, Desborough JP, Powell H, Burrin JM, Hall GM. Effects of extradukral anaesthesia on interleukin-6 abd acute phase response to surgery. Br J Anaest. 1994;72(3):272–9. 10.1093/bja/72.3.272 7510510

[18] Watt DG, Horgan PG, McMillan DC. Routine clinical markers of the magnitude of the systemic inflammatory response after elective operation: a systematic review. Surgery. 2015;157(2):362–80. 25616950 10.1016/j.surg.2014.09.009

[19] Desborough JP. The stress response to trauma and surgery. Br J Anesth. 2000;85(1):109–17. 10.1093/bja/85.1.109 10927999

[20] Kudoh A, Katagai H, Takazawa T. Plasma inflammtory cytokine response to surgical trauma in chronic depressed patients. Cytokine. 2001;13(2):104–8. 11145850 10.1006/cyto.2000.0802

[21] Gail MH, Wieand S, Piantadosi S. Biased estimates of treatment effect in randomized experiments with nonlinear regressions and omitted covariates. Biometrika. 1984;71(3):431–44. 10.1093/biomet/71.3.431. 10.1093/biomet/71.3.431

[22] Quispe MRF, Salgado JW. Transabdominal preperitoneal (TAPP) versus open Lichtenstein hernia repair: comparison of the systemic inflammatory response and the postoperative pain. Acta Cir Bras. 2019;34(2):e201900206. 30843939 10.1590/s0102-8650201900206 PMC6585912

[23] Cochran WG. Some methods for strengthening the common χ 2 tests. Biometrics. 1954;10(4):417. 10.2307/3001616. 10.2307/3001616

[24] Bender R, Lange S. Adjusting for multiple testing–when and how? J Clin Epidemiol. 2001;54(4):343–9. 10.1016/s0895-4356(00)00314-0. 11297884 10.1016/s0895-4356(00)00314-0

